# Short-Term Effects of Ambient Air Pollution on Hospitalization for Respiratory Disease in Taiyuan, China: A Time-Series Analysis

**DOI:** 10.3390/ijerph15102160

**Published:** 2018-10-01

**Authors:** Lisha Luo, Yunquan Zhang, Junfeng Jiang, Hanghang Luan, Chuanhua Yu, Peihong Nan, Bin Luo, Mao You

**Affiliations:** 1 Department of Preventive Medicine, School of Health Sciences, Wuhan University, Wuhan 430071, China; 13006362970@163.com (L.L.); Yun-quanZhang@whu.edu.cn (Y.Z.); jiang0111@whu.edu.cn (J.J.); 2 Department of Forensic Medicine, Tongji Medical College, Huazhong University of Science and Technology, Wuhan 430030, China; luanhh@hust.edu.cn; 3 Global Health Institute, Wuhan University, Wuhan 430071, China; 4 Institute of Health Administration, Shanxi, Taiyuan 030006, China; tgnph@163.com; 5 Ddata Technology Co. LTD, Wuhan 430000, China; luo-bin@dldata.cn; 6 National Health Development Research Center, Beijing 100191, China; ym@nhei.cn

**Keywords:** air pollution, hospitalization, respiratory disease, China

## Abstract

In this study, we estimated the short-term effects of ambient air pollution on respiratory disease hospitalization in Taiyuan, China. Daily data of respiratory disease hospitalization, daily concentration of ambient air pollutants and meteorological factors from 1 October 2014 to 30 September 2017 in Taiyuan were included in our study. We conducted a time-series study design and applied a generalized additive model to evaluate the association between every 10-μg/m^3^ increment of air pollutants and percent increase of respiratory disease hospitalization. A total of 127,565 respiratory disease hospitalization cases were included in this study during the present period. In single-pollutant models, the effect values in multi-day lags were greater than those in single-day lags. PM_2.5_ at lag02 days, SO_2_ at lag03 days, PM_10_ and NO_2_ at lag05 days were observed to be strongly and significantly associated with respiratory disease hospitalization. No significant association was found between O_3_ and respiratory disease hospitalization. SO_2_ and NO_2_ were still significantly associated with hospitalization after adjusting for PM_2.5_ or PM_10_ into two-pollutant models. Females and younger population for respiratory disease were more vulnerable to air pollution than males and older groups. Therefore, some effective measures should be taken to strengthen the management of the ambient air pollutants, especially SO_2_ and NO_2_, and to enhance the protection of the high-risk population from air pollutants, thereby reducing the burden of respiratory disease caused by ambient air pollution.

## 1. Introduction

As one of the chronic non-communicable diseases, respiratory disease has become the 4th leading cause of death globally, which has brought tremendous health burdens to society. According to the Global Burden of Disease study 2016 (GBD 2016), there were 3.54 million deaths and 92.53 million disability-adjusted life years (DALYs) caused by respiratory disease globally in 2016. In China, respiratory disease caused approximately 0.92 million deaths and 17.81 million DALYs in 2016, which ranked the third leading cause of death [1,2]. It has been shown that respiratory disease is caused by a variety of risk factors, among which tobacco use and air pollution are the most important risk factors [3].

With the rapid development of economy and industrialization, China has experienced a severe deterioration in air quality over the past several years. Air pollution was the fourth highest-ranking risk factor and led to approximately 1.58 million deaths and 32.28 million DALYs in 2016 [3]. Unlike the behavioral risk factors such as alcohol use and tobacco use, and metabolic risk factors such as high blood pressure and high blood glucose, ambient air pollution is one of the potentially modifiable environmental risk factors that do not depend on the change of individual behaviors [4]. In consequence, reducing concentration of air pollutants and strengthening individual protection can offer special advantages on respiratory disease prevention, thereby reducing the health and economic burden caused by respiratory disease from a public health perspective.

A growing number of studies had shown the association of short-term exposure to ambient air pollution and risk of respiratory disease in China [5,6,7,8,9]. Ren et al. assessed the association between air pollutants and respiratory disease mortality in Wuhan, China from 2007 to 2009 with both time-series and case-crossover methods, and they concluded that the increment in SO_2_ and NO_2_ were associated with an increase of relative risk for respiratory disease mortality [5]. Zhang et al. estimated the burden of years of life lost and mortality due to ambient PM_10_ pollution in Wuhan, China during 2009 to 2012, and they indicated that a 10-μg/m^3^ increment in PM_10_ was associated with an increase of 0.66% for respiratory disease mortality [6]. Based on a Generalized Additive Model (GAM), Tian et al. explored the relationship between PM_2.5_ and hospital visits for asthma in Beijing, China during 2010–2012 and concluded that the short-term elevations in PM_2.5_ concentration could increase the risk of asthma exacerbations [7]. Wang et al. analyzed the relationship between PM_10_ and cause-specific mortality from 2006 to 2010 in Tanggu, Tianjin, and their results showed that a 10-μg/m^3^ increase of PM_10_ was associated with a 0.88% increase in cardiopulmonary mortality [8]. Tao et al. studied the relationship between PM_10_, SO_2_, NO_2_ and respiratory disease hospitalization from 2001 to 2005 in Lanzhou, China, and stronger effects were observed for females and aged 65+ years groups [9].

Although these studies have observed the effects of different ambient air pollutants on risk of respiratory disease with different findings, their locations were limited to some large cities, and studies in recent five years have been still very sparse. As far as we know, the characteristic of air pollution varied in different regions and periods, and the health hazards were also inconsistent, which mainly depend on their unique development characteristics [10]. Taiyuan, the capital of Shanxi province, is the main area of coal production and rich in coal resources in China [11]. The type of air pollution in Taiyuan is typical coal-burning pollution, including particulate matter, sulfur dioxide and nitrogen oxide, which mainly come from the exhaust emissions of the heavy industries and coal-fired heating. One previous study showed that the particulate matter species in Taiyuan were different from those in other areas, and the concentration of heavy metals related to coal combustion in the chemical composition of particulate matter increased significantly, which indicated that coal combustion had a greater impact on the chemical composition of PM pollution. [12]. Therefore, the link between air pollution and respiratory disease in other areas could not directly reflect the status in Taiyuan. With the rapid development of the transportation, gas emission has also become one of the main sources of air pollution in Taiyuan [13]. Due to the specific geographical position and the climate type, ambient air pollution can hardly spread out immediately and the air condition is getting worse. Previous studies on the adverse effect of air pollutants on health in Taiyuan mainly focused on household air pollution, evidence-based epidemiologic researches linking ambient air pollution with respiratory disease have been still very sparse. Report on air quality in 74 cities by China National Environmental Monitoring Centre indicated that the air quality of Taiyuan ranked 69th from January to June 2017 based on the comprehensive index evaluation of urban environmental air quality [14]. Therefore, it is urgent to further investigate the effects of ambient air pollutants on respiratory disease in Taiyuan city, and to compare whether these results are consistent with studies in other cities.

Overall, this study has three advantages. First, as we mentioned above, PM species in Taiyuan were different from those in other areas due to the coal-burning pollution. Therefore, this study conducted in this understudied city benefited us to better understand the effect of the air pollution on respiratory disease in a coal-burning polluted region. Second, more recent data were used because the characteristic of air pollution varied in different periods. Third, the air pollution in our study consisted of five specific pollutants, so we could observe a relative full picture of the association between air pollution and respiratory disease.

In the present study, a time-series analysis was conducted to estimate the association of short-term exposure to air pollution and hospitalization due to respiratory disease and two subtypes (pneumonia and chronic obstructive pulmonary disease (COPD)) in Taiyuan with the latest data during 2014–2017. Gender and age were considered to examine whether health effect of air pollution differentiated between subgroups.

## 2. Materials and Methods

### 2.1. Study Area

Taiyuan (37°27′–38°25′ North, 111°30′–113°09′ East), the capital of Shanxi Province, is located in the east of the Yellow River valley of Northern China, which has a typical north temperate continental climate with four distinct seasons. Our study is limited to the urban districts of Taiyuan, with a total area of 1460 km^2^ and a population size of 3.41 million at the end of 2017.

### 2.2. Air Pollution and Meteorology Data

Eight air quality monitoring stations have been established in Taiyuan city by Taiyuan Environmental Protection Bureau. In the present study, daily average concentration of particulate matter less than 2.5 μm in aerodynamic diameter (PM_2.5_), particulate matter less than 10 μm in aerodynamic diameter (PM_10_), sulfur dioxide (SO_2_), nitrogen dioxide (NO_2_) and ozone (O_3_) between October 1st, 2014 and September 30th, 2017 were obtained from Taiyuan Environmental Protection Bureau. To adjust for the effects of weather on respiratory disease hospitalization, daily meteorological data including average temperature (°C) and relative humidity (%) during the same period were collected from the China Meteorological Data Network (http://data.cma.cn).

### 2.3. Hospitalization of Respiratory Disease

Data on daily hospitalization for respiratory disease from October 1st, 2014 to September 30th, 2017 were obtained from hospital electronic health records (EHRs) in 11 hospitals in urban districts of Taiyuan city. We extracted records including admission time, age, gender and the principle discharge diagnosis according to the International Classification of Diseases, 10th Revision (ICD-10) for each hospitalization. Hospitalization for respiratory disease (ICD-10: J00–J99) and two main categories: pneumonia (ICD-10: J18) and chronic obstructive pulmonary disease (COPD, ICD-10: J40–J44 and J47), were included into our study. We further classified the total respiratory disease hospitalization into different subgroups by gender (males and females) and age (0–64 years, 65+ years).

### 2.4. Data Analysis

Daily respiratory disease hospitalization, air pollutants and meteorological factors were described as mean ± standard deviation (SD) and quartiles. Spearman rank correlation analysis was conducted to estimate the relationships between air pollutants and meteorological factors. A time-series analysis approach in Generalized Additive Model (GAM) was applied to estimate the association between ambient air pollution and hospitalization for respiratory disease [15]. Since daily hospitalization counts typically followed an over-dispersed Poisson distribution, the quasi-Poisson distribution was adopted in the GAM model in our study [16,17,18]. The GAM process in the present study included the following steps: (1) The cubic spline smoothing function of time was incorporated into the GAM to control the long-term trends and seasonal changes of daily respiratory disease hospitalization [19]. (2) The partial autocorrelation function (PACF) was applied to select the degree of freedom of time according to the minimum absolute values of the sum of PACF for lags up to 30 [19,20]. Degrees of freedom for temperature and relative humidity were defined based on previous studies [9,21]. (3) Indicators for the weekends (DOW) and the public holidays (holiday) were adjusted as dummy variables in the model [6,19]. (4) The naturally smooth functions of two variables, average temperature and relative humidity, were included into the model to control the confounding effects of meteorological factors on the association between air pollution and respiratory disease hospitalization [21]. According to previous studies, the effect of temperature on health had a lag effect of more than 10 days [22,23]. Therefore, the 14-day moving average of mean temperature, and the relative humidity of the currant day were used in this study [6].

The model was as follows:

Log [E(Yt)] = α + s (time, df) + s (temperature, df) + s (humidity, df) + DOW+ holiday+ βZt

E(Yt) was the expected count of hospitalization for respiration disease on day t; α was the intercept of the model; s was a spline smoothing function for the nonlinear variables such as time, temperature and humidity; df was the degree of freedom and β denoted the coefficient for air pollutants of PM_2.5_, PM_10_, SO_2_, NO_2_ and O_3_; and Zt represented the concentration of each air pollutant on day t. In the final model, we defined 4 df per year for time trends, and 3 df per year for temperature and relative humidity.

Air pollutants of PM_2.5_, PM_10_, SO_2_, NO_2_ and O_3_ were incorporated into the model separately to evaluate the relationship between respiratory disease and each pollutant. Due to the potential delayed effects of ambient air pollutants on respiratory disease hospitalization [24], this study used the single-day lags (lag0, lag1, lag2, lag3, lag4, lag5) and multi-day lags (lag01, lag02, lag03, lag04, lag05) to estimate the effects of air pollutants at different lag days. Lag1 referred to the concentration of air pollutants at the previous day and lag01 meant the average concentration of air pollutants at the current and previous day. After evaluating the health effects of single-pollutant models, multi-pollutants models were also constructed to assess the stability of the effects of each air pollutants on the hospitalization for respiratory disease. Moreover, we analyzed the association between air pollutants and respiratory disease hospitalization in different subgroups by age (0–64 years and 65+ years) and gender (males and females). In addition to two- and multi-pollutants models, sensitivity analysis were conducted by varying dfs for time (2–5 per year), temperature and relative humidity (2–5) to examine the robustness of the results in our study (Table S1).

Consistent with previous studies [4,16], results in the present study were showed as percent changes and their 95% confidence intervals (CIs) in daily hospitalization for respiratory disease associated with per 10-μg/m^3^ increase in the concentration of air pollutants. All statistical analysis was carried out using R software (version 3.5.0, R Foundation for Statistical Computing, Vienna, Austria). Results with a 2-sided and *p* value < 0.05 were considered to be statistically significant.

## 3. Results

**Figure 1:**
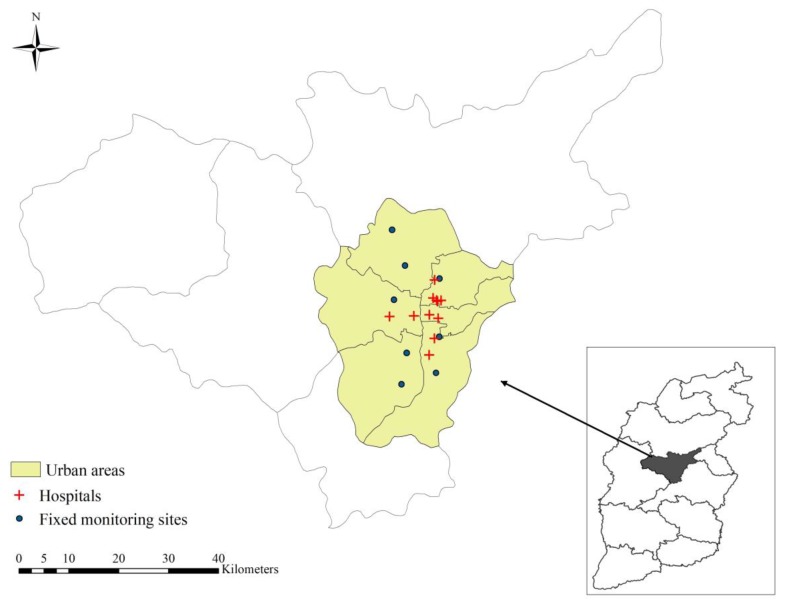
The spatial distributions of air monitoring sites and hospitals in Taiyuan, China.

The spatial distributions of air monitoring sites and hospitals are displayed in Figure 1. We observed that all of them were concentrated in the main urban areas of Taiyuan. The descriptive statistics for respiratory disease hospitalization, air pollutants and meteorological factors were summarized in Table 1. We included a total of 127,565 hospitalization cases with principle discharge diagnosis of respiratory disease from October 1st, 2014 to September 30th, 2017, of whom 78,209 (61.31%) were males and 49,356 (38.69%) were females. Daily mean hospitalization cases were 116.39 for total respiratory disease, among which 41.87 for pneumonia and 19.38 for COPD. For air pollutants, the daily average concentration for PM_2.5_, PM_10_, SO_2_, NO_2_, O_3_ were 65.71 μg/m^3^, 124.20 μg/m^3^, 69.34 μg/m^3^, 43.41 μg/m^3^ and 89.26 μg/m^3^, respectively. The average daily temperature was 11.39 °C, and average relative humidity was 57.70%.

**Figure 2:**
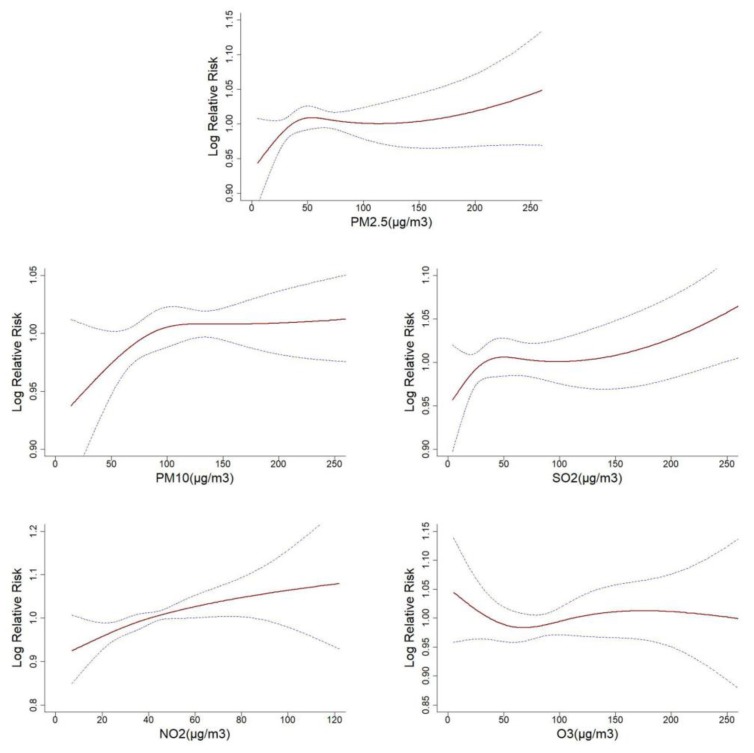
Exposure-response relationships between five air pollutants and respiratory disease hospitalization (The *x*-axis represented the concentration of air pollutants (μg/m^3^) at the current day, the *Y*-axis indicated Log relative risk of respiratory disease hospitalization. The blue imaginary lines were the 95% CI. All models were adjusted for time, temperature, relative humidity, weekend and public holiday.).

Figure 2 illustrated the exposure-response relationships between five air pollutants at the current day and the relative risk of respiratory disease hospitalization. The curves associated with PM_2.5_, PM_10_, SO_2_ and NO_2_ presented similar linear trends, which indicated that the higher concentration of air pollutants might cause a significant increase in the hospitalization for respiratory disease. In addition, O_3_ curve tended to be not associated with hospitalization. Similar exposure-response associations were identified in subtypes of pneumonia and COPD (Figure S3).

Table 2 showed the results of spearman rank correlation between air pollutants and meteorological factors. There were significantly positive relationships between PM_2.5_ and PM_10_, SO_2_, NO_2_, and the correlation coefficients (*r*) were 0.620–0.898 (*p* < 0.01). In accordance with PM_2.5_, PM_10_ also had strong correlations with SO_2_ and NO_2_ (*r* = 0.583 and 0.645, respectively, *p* < 0.01). The correlation coefficient between SO_2_ and NO_2_ was 0.496 (*p* < 0.01). Negative relationships were observed between O_3_ and other air pollutants. In addition, PM_2.5_, PM_10_, SO_2_ and NO_2_ had significantly negative correlations with temperature (*p* < 0.01), while these negative associations were just found between relative humidity and SO_2_.

The results of single-pollutant models at different lags are presented in Table 3. After adjusting for the confounding effects of meteorological factors, holiday and weekend, the effects of PM_2.5_, PM_10_, SO_2_ and NO_2_ on respiratory disease hospitalization had statistical significance (*p* < 0.05). For the single-day lag effects, the largest positive percent changes of respiratory disease hospitalization with a 10-μg/m^3^ increment in PM_2.5_ were found at lag0 with 0.397% (95%CI: 0.045–0.751), PM_10_ at lag0 with 0.257% (0.031–0.484), SO_2_ at lag1 with 0.413% (0.121–0.706) and NO_2_ at lag0 with 1.682% (0.664–2.711). In terms of multi-day lag effects, the strongest effects of PM_2.5_, PM_10_, SO_2_ and NO_2_ concentration on respiratory disease were 0.534% (0.092–0.977) at lag02, 0.389% (0.021–0.758) at lag05, 0.777% (0.320–1.237) at lag03 and 2.666% (0.961–4.399) at lag05, respectively. The effect values in single-day lags were smaller than those in multi-day lags. The increase in O_3_ concentration had no statistically significant effect on respiratory disease hospitalization, either for single- or multi-day lags.

After the best lag day for each pollutant being determined in the single-pollutant models, we added other pollutants for adjustment. The percent increase and 95% CI for respiratory disease hospitalization associated with a 10-μg/m^3^ increment of air pollutants in multi-pollutant models were presented in Table 4. After adjusting for O_3_ concentration in the two-pollutant models, the percent increase for respiratory disease hospitalization of other four pollutants appeared to be statistically significant, which was similar with the results of the single-pollutant models. Percent changes of PM_2.5_ and PM_10_ tended to be statistically insignificant adjusting for SO_2_ or NO_2_. On the contrary, although the values had decreased, percent increase of SO_2_ and NO_2_ were still statistically significant after adding PM_2.5_ or PM_10_ in two-pollutant models. When we added other four pollutants in the multi-pollutant models, all effect values appeared to be statistically insignificant.

The percent increase and 95% CI for hospitalization of different types of respiratory disease with a 10-μg/m^3^ increase in five air pollutants were listed in Table 5. After controlling the effects of meteorological and other factors, the estimated effects of PM_2.5_, PM_10_, SO_2_ and NO_2_ on pneumonia hospitalization were statistically significant at different lag days (*p* < 0.05). For PM_2.5_, the percent increase decreased gradually in single-day models, the largest values occurred at the current day (lag0) (0.541%, 95% CI: 0.187–0.897), while in multi-day models, the effect values increased with the lag days and the strongest effect was observed at lag05 (0.990%, 95% CI: 0.407–1.576). The variations of PM_10_ and NO_2_ effects were generally consistent with PM_2.5_, and the greatest effects appeared at lag0 (PM_10_: 0.393% (0.160–0.626), NO_2_: 1.452% (0.403–2.512)) in single-day models and lag05 (PM_10_: 0.834% (0.453–1.216), NO_2_: 3.113% (1.332–4.926)) in multi-day models, respectively. The largest effect of SO_2_ on pneumonia was observed at lag1 with 0.438% (0.149–0.729) in single-pollutant models and lag04 with 1.017% (0.520–1.516) in multi-pollutant models. The effect of increased concentration of O_3_ on the hospitalization for pneumonia was not statistically significant. However, the effects of five air pollutants on COPD hospitalization were different from pneumonia. We could see that the percent increase with per 10-μg/m^3^ increment in PM_2.5_ and PM_10_ was statistically significant at the current day only (PM_2.5_: 0.547% (0.038–1.058), PM_10_: 0.366% (0.042–0.691), respectively). For SO_2_ and NO_2_, statistically significant effects appeared at lag0 in single-day models and different lag days in multi-day models. Different from overall respiratory disease and pneumonia, the association between O_3_ concentration and COPD hospitalization had statistically significance at lag1 (1.080% (0.326–1.839)), lag01 (1.073% (0.164–1.990)) and lag02 (1.124% (0.128–2.130)), respectively.

**Figure 3:**
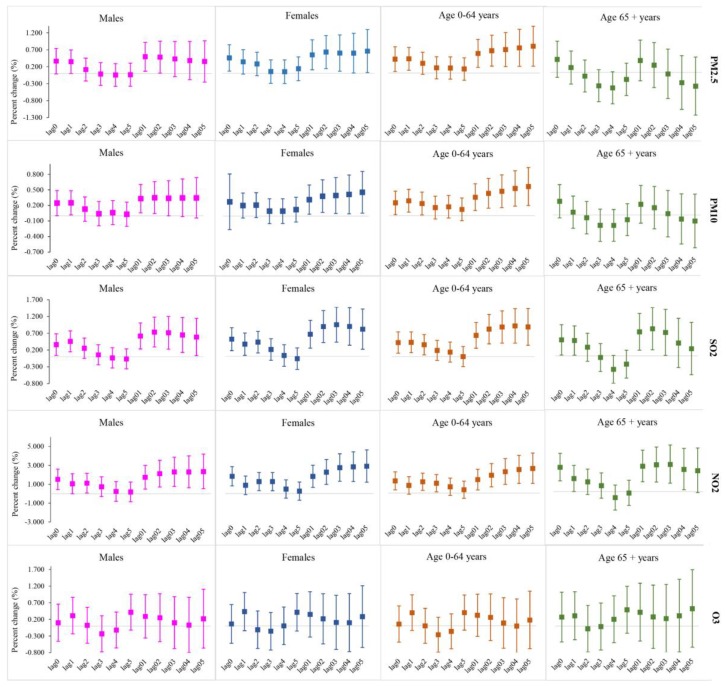
The percent change and 95% CI for hospitalization due to respiratory disease associated with a 10-μg/m^3^ increases in air pollutants concentrations by gender and age in single-pollutant models in Taiyuan, China.

The percent change of overall respiratory disease hospitalization with per 10-μg/m^3^ increment in single-pollutants by gender and age were shown in Figure 3. For gender subgroups, females suffered more from the adverse effects of air pollutants, with an increasing trend from lag01 to lag05 in multi-day lags. Except for SO_2_ at lag03 day, the significantly greatest effects values with PM_2.5_, PM_10_ and NO_2_ for female groups were observed at the current day in single-day lags and lag05 days in multi-day lags. For age subgroups, greater percent increase was displayed in younger groups aged 0–64 years, which meant they were more vulnerable to exposure to the air-pollutants, while in single-day lags, only the significant effects for SO_2_ and NO_2_ in group aged 65+ years at lag1 with 0.442% (0.021–0.865) and lag0 with 2.662% (1.172–4.173) were greater than those in groups aged 0–64 years. In accordance to overall respiratory disease, the associations between O_3_ concentration and hospitalization for age and gender subgroups had no statistical significance.

## 4. Discussion

In this study, we used the time-series design with Generalized Additive Model to analyze the relationships between air pollutants and hospitalization for respiratory diseases in Taiyuan, China. We concluded that after adjusting for influences of temperature, relative humidity, weekend and public holiday, PM_2.5_, PM_10_, SO_2_ and NO_2_ had statistically significant adverse effects on the hospitalization for overall respiratory disease and its subtypes, including pneumonia and COPD. Subgroups analysis demonstrated that females and younger groups aged 0–64 years were more vulnerable to air pollutants. Our results may have important implications for the prevention and treatment for respiratory disease and decision-making for governance of air pollution in Taiyuan, China.

The China National Environmental Monitoring Centre reported that, in the first half of 2017, the average concentration of PM_2.5_, PM_10_, SO_2_, NO_2_ and O_3_ of Taiyuan was 78, 147, 79, 55 and 185 μg/m^3^, ranked the 66th, 68th, 74th, 64th and 51th in 74 cities of China, respectively [14]. Therefore, it was of great significance and urgency to estimate the health burden caused by air pollution in Taiyuan.

Our findings showed that in single-day lags, a 10-μg/m^3^ increment in PM_2.5_ at lag0 was mostly strongly associated with 0.397% (95%CI: 0.045–0.751), 0.541% (0.187,0.897) and 0.547% (0.038,1.058) increment in overall respiratory, pneumonia and COPD hospitalization, respectively, and the effects in multi-day lags were greater than them, which were consistent with most previous studies [21,25,26]. A study in Jinan city conducted by Liu et al. concluded that an increase of 10-μg/m^3^ in PM_2.5_ corresponded to 1.4% (95% CI: 0.7–2.1) growth in respiratory emergency room visits in urban areas, and 1.5% (95%CI: 0.4–2.6) rise for suburban population [21]. A meta-analysis conducted by Li et al. yielded 12 studies to evaluate the association between exposure to PM_2.5_ and COPD hospitalization, and they found that a 10-μg/m^3^ increase in PM_2.5_ at lag0–7 days could lead to a 3.1% (1.6–4.6%) increase in COPD hospitalization [25]. However, Ouyang explored the effect of air pollution on pneumonia hospitalization in a children hospital in Changsha city, and they indicated that there was no significant association between PM_2.5_ and respiratory disease hospitalization [26], which was consistent with the study conducted by Rodopoulou et al. in Central Arkansas [16]. The results’ inconsistence is probably because Taiyuan has been exposed to a high concentration of pollution for a long time, and people's susceptibility is different from other areas [27]. As we mentioned above, the PM_2.5_ species in Taiyuan were also different from those in other areas because of the coal-burning. In addition, different study periods, the characteristics of included hospital and patients can also influence the results [6].

For PM_10_, we concluded that the increases of 0.257% (0.031, 0.484) at lag0 in single-day lags and 0.389% (0.021, 0.758) at lag05 in multi-day lags for respiratory disease hospitalization were mostly strongly due to per 10-μg/m^3^ growth in PM_10_ concentration. Our finding was also consistent with other previous studies [9,28]. Tao et al. concluded that with per inter-quartile range (IQR) increase in PM_10_, respiratory disease hospitalization was significantly increased by 2.4% (0.5–4.2) at lag4 day in Lanzhou, China [9]. Zhu et al. conducted a meta-analysis to estimate the relationship between PM_10_ and COPD hospitalization, and they observed that a 10-μg/m^3^ increase in PM_10_ was associated with 2.7% increment in COPD hospitalization [28]. However, other two studies conducted in Wuhan all showed that there was no significant association between PM_10_ concentration and respiratory mortality [5,6]. It was possible that the mortality and hospitalization are not the same indicators, and the concentration of pollutants and temperature in Taiyuan are also different from that in Wuhan, so the result of them are inconsistent.

SO_2_ is one of the major pollutants in the coal-smoking type of the air pollution [11,29]. As Taiyuan is rich in coal resources and mainly relies on coal for heating and cooking, SO_2_ pollution in Taiyuan is more serious than other cities in China. [11]. As we mentioned, average SO_2_ concentration in Taiyuan in the first half of 2017 was the highest among 74 cities in China. Our study revealed that the adverse effect of SO_2_ on respiratory disease was delayed and cumulative. The stronger significantly effect on overall respiratory disease hospitalization was observed at lag1 with 0.413% (0.121–0.706) in single-day lags and 0.777% (0.320–1.237) at lag03 in multi-day lags, and the results for pneumonia, COPD were consistent with them. A study conducted in Lanzhou showed that respiratory disease hospitalization increased by 3.4% (0.2–6.7) at lag1 with per IQR increase in SO_2_ concentration during 2001–2005. Their subtypes analysis in this study also supported our results. [9]. A study in Guangzhou revealed that with per 10-μg/m^3^ increment in SO_2_, the significant adverse effects on respiratory emergency room visits were 1.07% (0.54, 1.60) and 1.16% (0.61, 1.70) increases at lag1 and lag3 in multi-pollutant model, separately [30]. In Jinan, a 10-μg/m^3^ increase in SO_2_ concentration was associated with 1.2% (0.5–1.9) and 0.8% (−0.7–2.3) increases of hospital emergency room visits for respiratory disease in urban and suburban areas, respectively [21]. All these studies conducted in different regions in China indicated that there were significant relationships between respiratory diseases and SO_2_ at different lag days. It mainly because SO_2_ has an immediate stimulation effect on respiratory mucosa, causing acute attacks to respiratory systems [31]. Consequently, some effective measures must be taken to strengthen the governance of SO_2_ pollution and the prevention of its adverse effect in Taiyuan.

Of the five pollutants included in this study, NO_2_ had the greatest adverse effect on hospitalization for respiratory disease. We observed that an increment of 1.682% (0.664–2.711) at lag0 and 2.666% (0.961–4.399) at lag05 were mostly strongly associated with per 10-μg/m^3^ increase in NO_2_. In single-day lags, the effect of NO_2_ on pneumonia was higher than that on COPD, while in multi-day lags, the greatest effect was found at lag05, which was lower than that on COPD at lag03. This finding was consistent with previous studies [9,16,25]. According to the rankings mentioned above, the NO_2_ pollution in Taiyuan was also very serious, and it was confirmed by toxicological studies that NO_2_ could increase the susceptibility of respiratory patients. Therefore, much more attention should be paid to NO_2_ pollution for public health in Taiyuan. Our study showed that there was no association between O_3_ and hospitalization for overall respiratory disease and pneumonia, but a significant relationship between O_3_ and COPD hospitalization was observed, which were entirely consistent with other previous studies [25,32]. Our study indicated that in two-pollutant models, the effects of SO_2_ and NO_2_ were still statistically significant after adjusting for PM_2.5_ or PM_10_, whereas the effects of PM_10_ and PM_2.5_ were not significant when SO_2_ or NO_2_ were controlled, which meant SO_2_ and NO_2_ might be strong predictors for respiratory disease hospitalization in Taiyuan, China [33]. However, no significant association between air pollutants and hospitalization for respiratory disease was observed in multi-pollutant models. The possible reason was that the stronger correlations between air pollutants would influence the effects of them on respiratory disease hospitalization [18].

In line with previous studies, the subgroup analysis in our study indicated that females were more vulnerable to ambient air pollution, which might be due to the biological susceptibility. Unlike males, females have smaller lung tissue and trachea, which would be subjected to great pressure under the same pollution conditions [21,34]. Age-specific analysis revealed that the younger population (aged 0–64 years) were more sensitive to ambient pollution. The results of studies on age differences in the effects of air pollution were inconsistent, and most studies showed that older people were more sensitive to ambient air pollution because of their poor immune function [6,9]. While a small number of studies confirmed that younger population were more vulnerable than the elderly, which was consistent with our study [33]. The major reason was the time of exposure to ambient air pollution. Younger population spend more time outside because of work, learning, social interaction and so on, which obviously increase the exposure to air pollutants. In addition, younger people are more likely to expose themselves to the risk factors of respiratory disease, such as smoking and alcohol use [33,35].

Nevertheless, our study had several limitations. Firstly, as in previous studies, the data of ambient air pollutants and meteorological factors in this study were obtained from eight fixed monitoring stations in Taiyuan, which could not represent total exposure to population. Therefore, our study might underestimate the effects of these five pollutants on respiratory disease hospitalization. Secondly, we didn’t estimate the effects of air pollutants on asthma due to the data sparsity. However, some previous studies had shown that there was a significant association between asthma and ambient air pollution [7,36], so further epidemiological studies were needed to confirm this relationship in Taiyuan. Thirdly, the personal exposure to air pollution included ambient and indoor pollution, while our study lacked analysis of latter because of the unavailability of data, which would overestimate the effect of air pollution on respiratory disease. In addition, we didn’t include other meteorological factors like atmosphere pressure and wind speed into models, because there was no evidence supporting the associations between respiratory disease and them. Also, our study didn’t include the factors like marital status, occupation and education status because these data were not available. Further researches on these potential effects on respiratory disease hospitalization attributed to air pollution need to be conducted in Taiyuan.

## 5. Conclusions

In summary, we evaluated the short-term effects of ambient air pollutants on hospitalization for respiratory disease during 2014–2017 in Taiyuan. Through the Generalized Addition Model of time-series analysis, we observed positive associations between PM_2.5_, PM_10_, SO_2_ and NO_2_ concentration at different lag days and hospitalization for overall respiratory disease, pneumonia and COPD. NO_2_ and SO_2_ were the largest two risk pollutants of respiratory disease hospitalization in Taiyuan. Females and younger population for respiratory disease were more vulnerable to air pollution. In consequence, some effective measures should be taken to strengthen the management of the ambient air pollutants, especially SO_2_ and NO_2_, and to enhance the protection of the high-risk population from air pollutants, thereby reducing the burden of respiratory disease caused by ambient air pollution in Taiyuan.

## Figures and Tables

**Table 1:** Descriptive statistics for respiratory disease hospitalization, air pollutants and meteorological factors in Taiyuan, China, 2014–2017.

Factors	Mean ± SD	Min	P_25_	Median	P_75_	Max
Meteorological factors						
Temperature (°C)	11.39 ± 10.13	−8.06	1.02	12.53	21.07	27.54
Relative humidity (%)	57.70 ± 17.53	14	43	59	72	96
Air pollutants						
PM_2.5_ (μg/m^3^)	65.71 ± 48.80	5.10	34.25	53.05	80.65	377.00
PM_10_ (μg/m^3^)	124.2 ± 68.37	13.70	75.93	113.80	151.00	561.00
SO_2_ (μg/m^3^)	69.34 ± 68.27	4.00	21.00	42.40	93.75	428.80
NO_2_ (μg/m^3^)	43.41 ± 17.32	7.00	32.00	41.35	52.68	122.00
O_3_ (μg/m^3^)	89.26 ± 53.47	5.00	47.00	79.50	124.75	275.00
Respiratory disease						
All	116.39 ± 47.36	20	79	115	148	255
Male	71.36 ± 29.53	12	49	71	91	171
Female	45.03 ± 19.04	6	30	44	57	109
0–64 years	85.18 ± 33.03	16	61	83	106	182
65+ years	31.21 ± 16.70	1	16	32	42	98
Pneumonia	41.87 ± 20.28	4	27	38	53	111
COPD	19.38 ± 10.48	1	11	19	26	62

**Table 2:** The coefficient of spearman rank correlation between air pollutants and meteorological factors in Taiyuan, China.

Factors	PM_2.5_	PM_10_	SO_2_	NO_2_	O_3_	RHU	TEM
PM_2.5_	1						
PM_10_	0.898 ***	1					
SO_2_	0.656 ***	0.583 ***	1				
NO_2_	0.620 ***	0.645 ***	0.496 ***	1			
O_3_	−0.253 ***	−0.132 ***	−0.611 ***	−0.073 *	1		
RHU	0.197 ***	0.028	−0.180 ***	0.161 ***	−0.002	1	
TEM	−0.327 ***	−0.243 ***	−0.755 ***	−0.114 ***	0.780 ***	0.361 ***	1

(Note: RHU: Relative Humidity; TEM: Temperature; * *p* < 0.05, ** *p* < 0.01, *** *p* < 0.001).

**Table 3:** The percent change and 95% CI for respiratory disease hospitalization associated with a 10-μg/m^3^ increases in air pollutants concentrations in single-pollutant models.

Lag Days	PM_2.5_	PM_10_	SO_2_	NO_2_	O_3_
lag0	0.397(0.045,0.751) *	0.257(0.031,0.484) *	0.407(0.106,0.708) **	1.682(0.664,2.711) **	0.088(−0.438,0.618)
lag1	0.342(0.017,0.668) *	0.232(0.012,0.452) *	0.413(0.121,0.706) **	1.018(0.020,2.026) *	0.359(−0.160,0.880)
lag2	0.176(−0.139,0.491)	0.160(−0.060,0.381)	0.309(0.026,0.593) *	1.228(0.249,2.217) *	−0.028(−0.538,0.485)
lag3	0.008(−0.307,0.325)	0.061(−0.160,0.282)	0.107(−0.175,0.389)	0.994(0.014,1.984) *	−0.203(−0.713,0.310)
lag4	−0.010(−0.326,0.307)	0.074(−0.144,0.292)	−0.016(−0.298,0.267)	0.344(−0.633,1.331)	−0.071(−0.580,0.441)
lag5	0.029(−0.289,0.348)	0.065(−0.154,0.284)	−0.069(−0.352,0.214)	0.219(−0.757,1.204)	0.415(−0.095,0.929)
lag01	0.512(0.113,0.912) *	0.326(0.068,0.584) *	0.617(0.254,0.981) ***	1.825(0.649,3.016) **	0.315(−0.300,0.934)
lag02	0.534(0.092,0.977) *	0.363(0.075,0.652) *	0.771(0.354,1.189) ***	2.255(0.939,3.589) ***	0.240(−0.440,0.924)
lag03	0.489(0.004,0.977) *	0.360(0.043,0.678) *	0.777(0.320,1.237) ***	2.574(1.128,4.041) ***	0.105(−0.629,0.846)
lag04	0.459(−0.070,0.991)	0.373(0.031,0.717) *	0.715(0.223,1.209) **	2.612(1.039,4.209) **	0.062(−0.724,0.853)
lag05	0.465(−0.107,1.040)	0.389(0.021,0.758) *	0.645(0.119,1.173) *	2.666(0.961,4.399) **	0.248(−0.589,1.092)

(Note: * *p *< 0.05, ** *p *< 0.01, *** *p *< 0.001).

**Table 4:** The percent change and 95% CI for respiratory disease hospitalization associated with a 10-μg/m^3^ increases in air pollutants concentrations at different best lag days in multi-pollutant models.

Variable	PM_2.5_	PM_10_	SO_2_	NO_2_	O_3_
Adjusted for PM_2.5_	-	0.136(−0.401,0.676)	0.762(0.116,1.413) *	2.188(0.182,4.233) *	0.409(−0.101,0.921)
Adjusted for PM_10_	0.414(−0.233,1.065)	-	0.718(0.183,1.256) **	2.613(0.345,4.931) *	0.381(−0.130,0.895)
Adjusted for SO_2_	0.020(−0.599,0.642)	0.091(−0.336,0.520)	-	1.552(−0.462,3.608)	0.434(−0.075,0.944)
Adjusted for NO_2_	0.229(−0.292,0.752)	0.017(−0.471,0.508)	0.541(−0.007,1.091)	-	0.363(−0.146,0.875)
Adjusted for O_3_	0.530(0.089,0.973) *	0.371(0.002,0.740) *	0.785(0.327,1.244) ***	2.583(0.877,4.319) **	-
Adjusted for other 4 pollutants	−0.021(−0.799,0.764)	−0.119(−0.733,0.498)	0.618(−0.065,1.305)	1.713(−0.674,4.157)	0.406(−0.104,0.919)

(Note: The best lag day was the day with the highest effect value in single-pollutant models, specifically for PM_2.5_ at lag02, PM_10_ at lag05, SO_2_ at lag03, NO_2_ at lag 05 and O_3_ at lag5. * *p *< 0.05, ** *p *< 0.01, *** *p *< 0.001).

**Table 5:** The percent change and 95% CI for hospitalization due to subtypes of respiratory disease associated with a 10-μg/m^3^ increases in air pollutants concentrations in single-pollutant models.

Disease	PM_2.5_	PM_10_	SO_2_	NO_2_	O_3_
Pneumonia					
lag0	0.541(0.187,0.897) **	0.393(0.160,0.626) ***	0.400(0.102,0.699) **	1.452(0.403,2.512) **	0.073(−0.524,0.673)
lag1	0.442(0.116,0.769) **	0.332(0.107,0.558) **	0.438(0.149,0.729) **	0.929(−0.096,1.966)	0.267(−0.321,0.859)
lag2	0.352(0.037,0.668) *	0.326(0.102,0.550) **	0.372(0.091,0.655) **	1.211(0.203,2.229) *	−0.04(−0.620,0.543)
lag3	0.259(−0.056,0.576)	0.268(0.044,0.493) *	0.248(−0.033,0.530)	1.279(0.266,2.303) *	−0.209(−0.789,0.375)
lag4	0.215(−0.102,0.532)	0.290(0.067,0.514) *	0.215(−0.066,0.496)	1.013(0.000,2.037)	−0.247(−0.824,0.334)
lag5	0.068(−0.251,0.389)	0.150(−0.075,0.376)	0.010(−0.272,0.292)	0.326(−0.680,1.343)	0.220(−0.359,0.803)
lag01	0.685(0.283,1.089) ***	0.485(0.221,0.751) ***	0.634(0.273,0.996) ***	1.615(0.404,2.840) **	0.240(−0.461,0.945)
lag02	0.797(0.352,1.244) ***	0.594(0.298,0.891) ***	0.835(0.420,1.251) ***	2.073(0.717,3.447) **	0.173(−0.603,0.956)
lag03	0.884(0.393,1.377) ***	0.682(0.356,1.008) ***	0.939(0.480,1.401) ***	2.580(1.084,4.098) ***	0.045(−0.797,0.894)
lag04	0.968(0.432,1.507) ***	0.788(0.435,1.143) ***	1.017(0.520,1.516) ***	2.987(1.351,4.649) ***	−0.083(−0.985,0.828)
lag05	0.990(0.407,1.576) ***	0.834(0.453,1.216) ***	0.984(0.450,1.521) ***	3.113(1.332,4.926) ***	0.023(−0.940,0.996)
COPD					
lag0	0.547(0.038,1.058) *	0.366(0.042,0.691) *	0.616(0.186,1.047) **	2.807(1.302,4.334) ***	0.367(−0.400,1.140)
lag1	0.249(−0.217,0.718)	0.132(−0.183,0.449)	0.382(−0.034,0.800)	1.267(−0.172,2.726)	1.080(0.326,1.839) **
lag2	−0.001(−0.451,0.451)	0.001(−0.316,0.319)	0.161(−0.240,0.564)	1.004(−0.399,2.426)	0.442(−0.297,1.188)
lag3	−0.039(−0.489,0.412)	−0.023(−0.339,0.294)	0.246(−0.148,0.642)	1.614(0.203,3.045) *	0.017(−0.725,0.765)
lag4	−0.330(−0.783,0.125)	−0.220(−0.533,0.095)	−0.203(−0.601,0.197)	−0.179(−1.572,1.233)	−0.164(−0.902,0.580)
lag5	−0.110(−0.566,0.348)	−0.045(−0.359,0.271)	−0.078(−0.476,0.321)	0.256(−1.142,1.673)	0.417(−0.325,1.164)
lag01	0.537(−0.037,1.115)	0.328(−0.042,0.698)	0.764(0.247,1.285) **	2.745(1.022,4.498) **	1.073(0.164,1.990) *
lag02	0.437(−0.197,1.076)	0.274(−0.140,0.690)	0.796(0.209,1.386) **	2.846(0.935,4.794) **	1.124(0.128,2.130) *
lag03	0.373(−0.323,1.075)	0.235(−0.220,0.692)	0.883(0.248,1.522) **	3.438(1.341,5.578) **	0.973(−0.096,2.054)
lag04	0.169(−0.589,0.933)	0.111(−0.381,0.605)	0.714(0.038,1.394) *	3.117(0.848,5.436) **	0.812(−0.329,1.967)
lag05	0.105(−0.713,0.931)	0.085(−0.442,0.615)	0.653(−0.064,1.375)	3.173(0.720,5.687) *	0.961(−0.251,2.188)

(Note: * *p *< 0.05, ** *p *< 0.01, *** *p *< 0.001).

## Supplementary Materials

The following are available online at http://www.mdpi.com/1660-4601/15/10/2160/s1, Figure S1: Boxplots of monthly hospitalization due to respiratory disease by gender and age in Taiyuan, China during 2014–2017. Figure S2: Time series of PM_2.5_, PM_10_, SO_2_, NO_2_ and O_3_ in Taiyuan, China, during 1st October 2014 to 30th September 2017. Figure S3: The exposure-response associations between five air pollutants and hospitalization for pneumonia and COPD. (The *x*-axis represented the concentration of air pollutants (μg/m^3^) at the current day, the *Y*-axis indicated Log relative risk of respiratory disease hospitalization. The blue lines were the 95% CI. All models were adjusted for time, temperature, relative humidity, weekend and public holiday.) Table S1: The percent change and 95% CI for hospitalization due to respiratory disease associated with a 10-μg/m^3^ increases in air pollutants concentrations by gender and age in single-pollutant models in Taiyuan, China. Table S2: Sensitivity analysis for health effects of ambient air pollution on respiratory disease hospitalization by changing df = 2–5 per year of time, temperature and relative humidity in Taiyuan, China (Health effects are shown as percent changes and 95% CI with per 10-μg/m^3^ increment in air pollutants).

## References

[1] Naghavi M., Abajobir A.A., Abbafati C., Abbas K.M., Abd-Allah F., Abera S.F., Aboyans V., Adetokunboh O., Afshin A., Agrawal A. (2017). Global, regional, and national age-sex specific mortality for 264 causes of death, 1980–2016: A systematic analysis for the Global Burden of Disease Study 2016. Lancet.

[2] Hay S.I., Abajobir A.A., Abate K.H., Abbafati C., Abbas K.M., Abd-Allah F., Abdulkader R.S., Abdulle A.M., Abebo T.A., Abera SF. (2017). Global, regional, and national disability-adjusted life-years (DALYs) for 333 diseases and injuries and healthy life expectancy (HALE) for 195 countries and territories, 1990–2016: A systematic analysis for the Global Burden of Disease Study 2016. Lancet.

[3] Gakidou E., Afshin A., Abajobir A.A., Abate K.H., Abbafati C., Abbas K.M., Abd-Allah F., Abdulle A.M., Abera S.F., Aboyans V. (2017). Global, regional, and national comparative risk assessment of 84 behavioural, environmental and occupational, and metabolic risks or clusters of risks, 1990–2016: A systematic analysis for the Global Burden of Disease Study 2016. Lancet.

[4] Liu H., Tian Y., Xiang X., Sun K., Juan J., Song J., Cao Y., Xu B., Hu Y. (2017). Air Pollution and Hospitalization for Acute Myocardial Infarction in China. Am. J. Cardiol..

[5] Ren M., Li N., Wang Z., Liu Y., Chen X., Chu Y., Li X., Zhu Z., Tian L., Xiang H. (2017). The short-term effects of air pollutants on respiratory disease mortality in Wuhan, China: Comparison of time-series and case-crossover analyses. Sci. Rep..

[6] Zhang Y., Peng M., Yu C., Zhang L. (2017). Burden of mortality and years of life lost due to ambient PM_10_ pollution in Wuhan, China. Environ. Pollut..

[7] Tian Y., Xiang X., Juan J., Sun K., Song J., Cao Y., Hu Y. (2017). Fine particulate air pollution and hospital visits for asthma in Beijing, China. Environ. Pollut..

[8] Wang T., Li G.X., Sun J., Buys N., Liu H.M., Liu M.F., Ni M., Li B.W., Liang X.F., Pan X. (2013). Association between ambient particulate matter and daily cause-specific mortality in Tanggu, Tianjin Binhai New Area, China. Int. J. Environ. Health Res..

[9] Tao Y., Mi S., Zhou S., Wang S., Xie X. (2014). Air pollution and hospital admissions for respiratory diseases in Lanzhou, China. Environ. Pollut..

[10] Chen R., Peng R.D., Meng X., Zhou Z., Chen B., Kan H. (2013). Seasonal variation in the acute effect of particulate air pollution on mortality in the China Air Pollution and Health Effects Study (CAPES). Sci. Total Environ..

[11] Tang D., Wang C., Nie J., Chen R., Niu Q., Kan H., Chen B., Perera F. (2014). Health benefits of improving air quality in Taiyuan, China. Environ. Int..

[12] Cao L., Geng H., Yao C., Zhao L., Duan P., Xuan Y., Li H. (2014). Investigation of chemical compositions of atmospheric fine particles during a wintertime haze episode in Taiyuan City. China Environ. Sci..

[13] Han F., Cao J., Peng L., Bai H., Hu D., Mu L., Liu X. (2015). Characteristics of hopanoid hydrocarbons in ambient PM_10_ and motor vehicle emissions and coal ash in Taiyuan, China. Environ. Geochem. Health.

[14] China National Environmental Monitoring Centre Air Quality Report in the First Half of 2017. http://www.cnemc.cn/kqzlzkbgyb2092938.jhtml.

[15] Xiong Q., Zhao W., Gong Z., Zhao W., Tang T. (2015). Fine Particulate Matter Pollution and Hospital Admissions for Respiratory Diseases in Beijing, China. Int. J. Environ. Res. Public Health.

[16] Rodopoulou S., Samoli E., Chalbot M.G., Kavouras I.G. (2015). Air pollution and cardiovascular and respiratory emergency visits in Central Arkansas: A time-series analysis. Sci. Total Environ..

[17] Burnett R.T., Smith-Doiron M., Stieb D., Cakmak S., Brook J.R. (1999). Effects of particulate and gaseous air pollution on cardiorespiratory hospitalizations. Arch. Environ. Health.

[18] Zhu J., Zhang X., Zhang X., Dong M., Wu J., Dong Y., Chen R., Ding X., Huang C., Zhang Q. (2017). The burden of ambient air pollution on years of life lost in Wuxi, China, 2012–2015: A time-series study using a distributed lag non-linear model. Environ. Pollut..

[19] Ma Y., Zhang H., Zhao Y., Zhou J., Yang S., Zheng X., Wang S. (2017). Short-term effects of air pollution on daily hospital admissions for cardiovascular diseases in western China. Environ. Sci. Pollut. Res..

[20] Ma Y., Zhao Y., Yang S., Zhou J., Xin J., Wang S., Yang D. (2017). Short-term effects of ambient air pollution on emergency room admissions due to cardiovascular causes in Beijing, China. Environ. Pollut..

[21] Liu P., Wang X., Fan J., Xiao W., Wang Y. (2016). Effects of Air Pollution on Hospital Emergency Room Visits for Respiratory Diseases: Urban-Suburban Differences in Eastern China. Int. J. Environ. Res. Public Health.

[22] Phung D., Thai P.K., Guo Y., Morawska L., Rutherford S., Chu C. (2016). Ambient temperature and risk of cardiovascular hospitalization: An updated systematic review and meta-analysis. Sci. Total Environ..

[23] Ma W., Chen R., Kan H. (2014). Temperature-related mortality in 17 large Chinese cities: How heat and cold affect mortality in China. Environ. Res..

[24] Pun V.C., Tian L., Yu I.T., Kioumourtzoglou M.A., Qiu H. (2015). Differential distributed lag patterns of source-specific particulate matter on respiratory emergency hospitalizations. Environ. Sci. Technol..

[25] Li M.H., Fan L.C., Mao B., Yang J.W., Choi A., Cao W.J., Xu J.F. (2016). Short-term Exposure to Ambient Fine Particulate Matter Increases Hospitalizations and Mortality in COPD: A Systematic Review and Meta-analysis. Chest.

[26] Ouyang F., Liu S., Mao J., Zheng Q., Ma T., Hu M. (2017). Relationship between air pollution and the number of pneumonia hospitalization in a children’s hospital in Changsha. Zhong Nan Da Xue Xue Bao Yi Xue Ban.

[27] Burte E., Nadif R., Jacquemin B. (2016). Susceptibility Factors Relevant for the Association Between Long-Term Air Pollution Exposure and Incident Asthma. Curr. Environ. Health Rep..

[28] Zhu R., Chen Y., Wu S., Deng F., Liu Y., Yao W. (2013). The relationship between particulate matter (PM_10_) and hospitalizations and mortality of chronic obstructive pulmonary disease: A meta-analysis. COPD.

[29] Li Y.R., Gibson J.M. (2014). Health and air quality benefits of policies to reduce coal-fired power plant emissions: A case study in North Carolina. Environ. Sci. Technol..

[30] Zhang Z., Wang J., Chen L., Chen X., Sun G., Zhong N., Kan H., Lu W. (2014). Impact of haze and air pollution-related hazards on hospital admissions in Guangzhou, China. Environ. Sci. Pollut. Res..

[31] Dong G.H., Zhang P., Sun B., Zhang L., Chen X., Ma N., Yu F., Guo H., Huang H., Lee Y.L. (2012). Long-term exposure to ambient air pollution and respiratory disease mortality in Shenyang, China: A 12-year population-based retrospective cohort study. Respiration.

[32] Ghozikali M.G., Mosaferi M., Safari G.H., Jaafari J. (2015). Effect of exposure to O_3_, NO_2_, and SO_2_ on chronic obstructive pulmonary disease hospitalizations in Tabriz, Iran. Environ. Sci. Pollut. Res..

[33] Zhang C., Ding R., Xiao C., Xu Y., Cheng H., Zhu F., Lei R., Di D., Zhao Q., Cao J. (2017). Association between air pollution and cardiovascular mortality in Hefei, China: A time-series analysis. Environ. Pollut..

[34] Oiamo T.H., Luginaah I.N. (2013). Extricating sex and gender in air pollution research: A community-based study on cardinal symptoms of exposure. Int. J. Environ. Res. Public Health.

[35] van Gemert F., Chavannes N., Kirenga B., Jones R., Williams S., Tsiligianni I., Vonk J., Kocks J., de Jong C., van der Molen T. (2016). Socio-economic factors, gender and smoking as determinants of COPD in a low-income country of sub-Saharan Africa: FRESH AIR Uganda. NPJ Prim. Care Respir. Med..

[36] Guarnieri M., Balmes J.R. (2014). Outdoor air pollution and asthma. Lancet.

