# Supplementary material for: Short-Term Effects of Ambient Air Pollution on Hospitalization for Respiratory Disease in Taiyuan, China: A Time-Series Analysis
Source: Int J Environ Res Public Health. 2018 Oct 1;15(10):2160. doi: 10.3390/ijerph15102160 (PMC6210308; doi:10.3390/ijerph15102160)
Supplement: Supplementary file 1 [file ijerph-15-02160-s001.pdf]

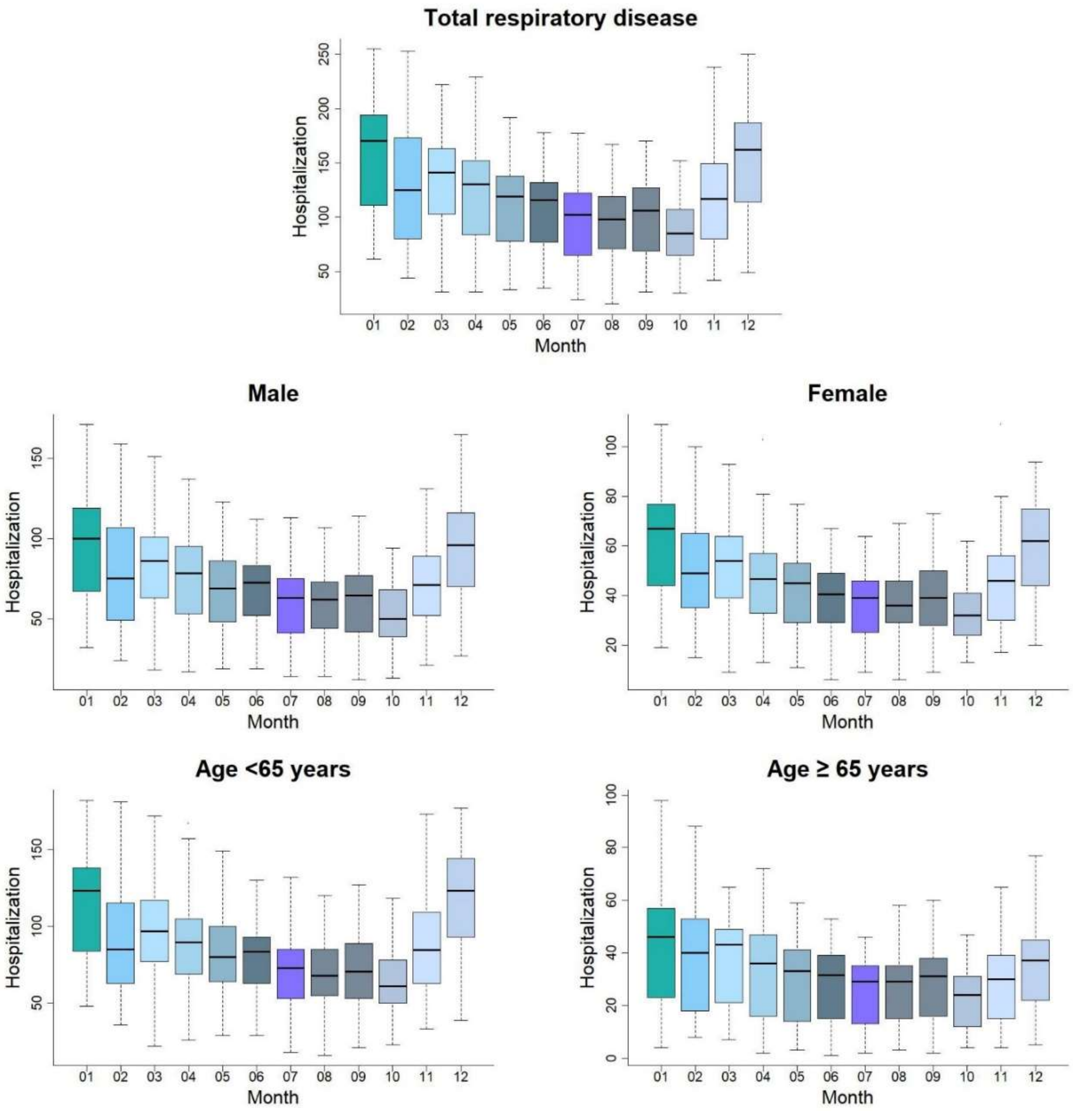

Figure S1. Boxplots of monthly hospitalization due to respiratory disease by gender and age in Taiyuan, China during 2014–2017.

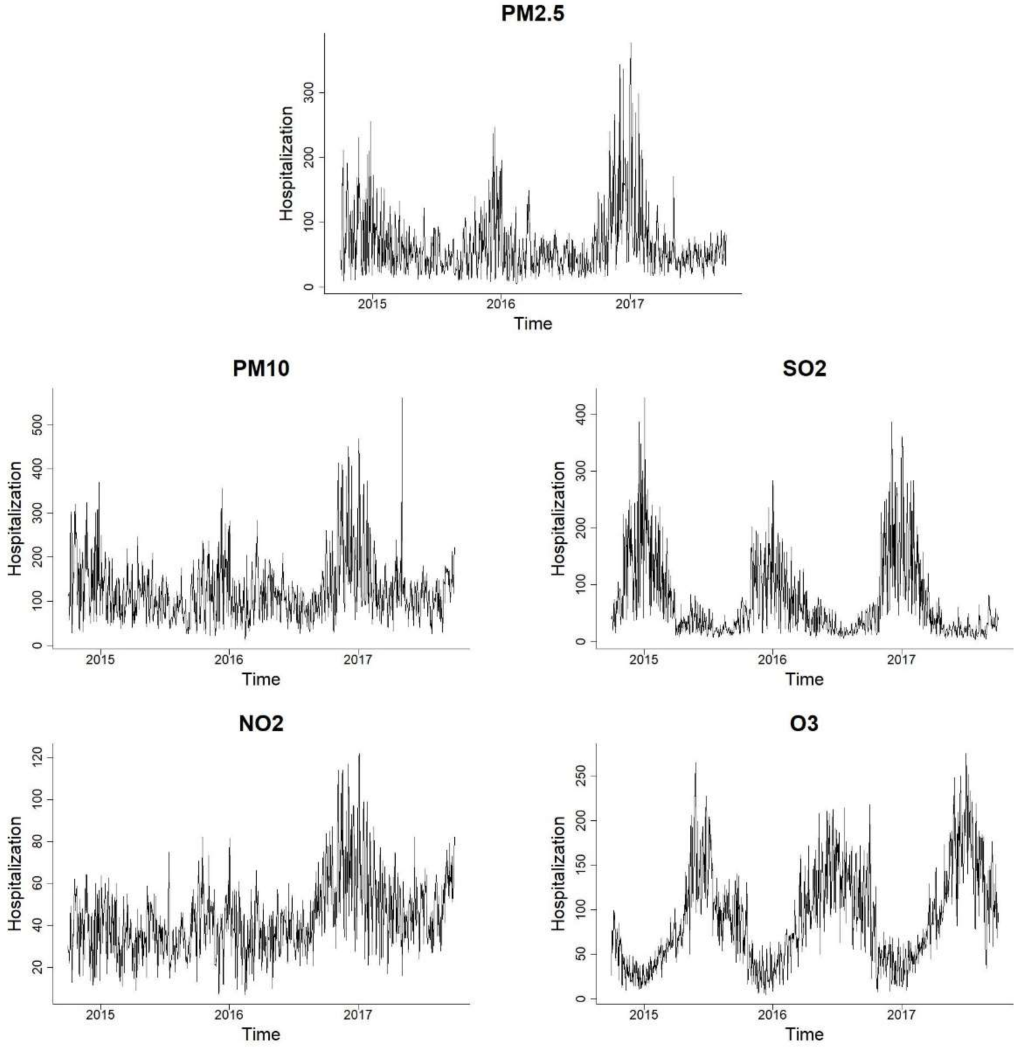

Figure S2. Time series of $PM_{2.5}$, PM_{10}, SO_2, NO_2 and O_3 in Taiyuan, China, during 1st October 2014 to 30th September 2017.

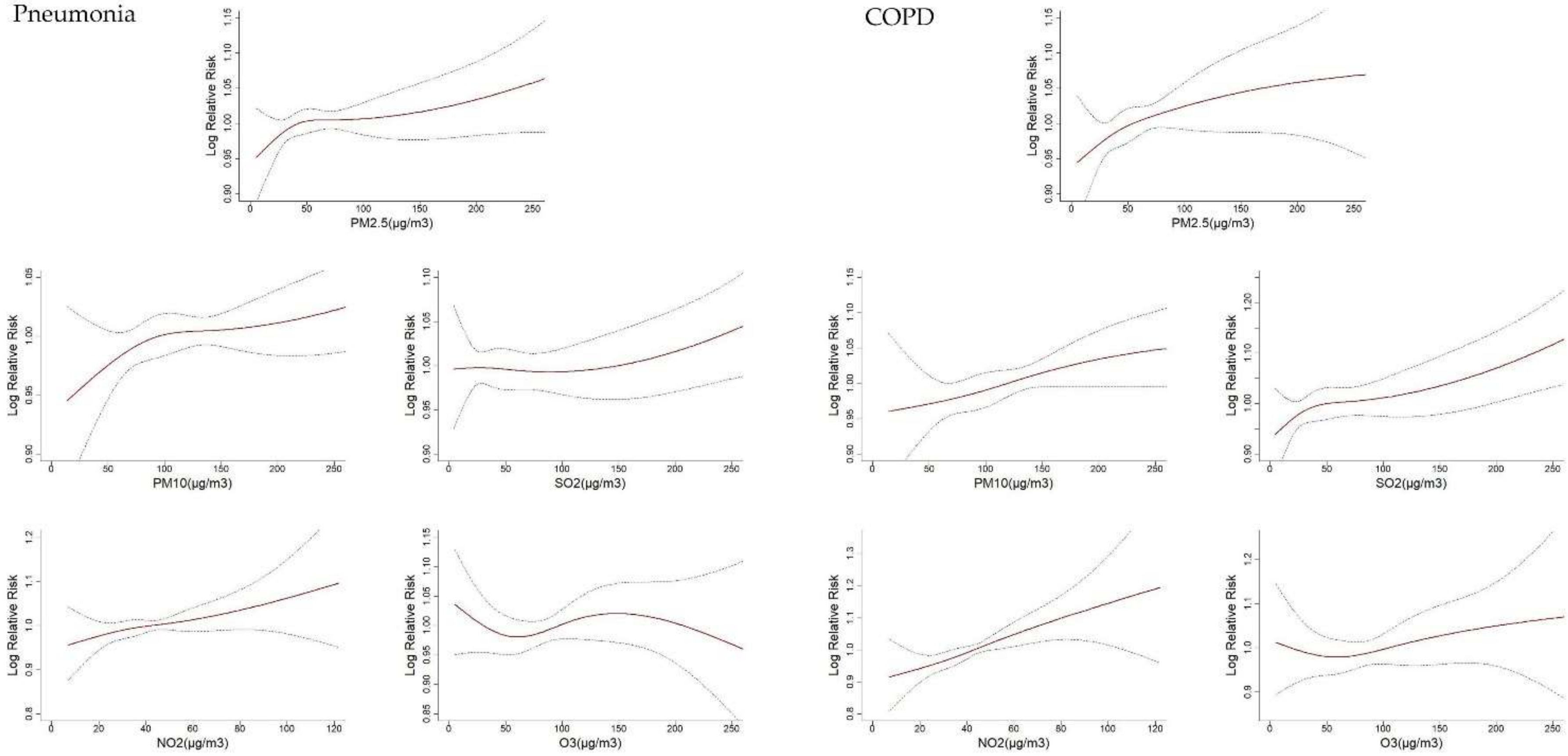

Figure S3. The exposure-response associations between five air pollutants and hospitalization for pneumonia and COPD. (The x-axis represented the concentration of air pollutants (ug/m3) at the current day, the Y-axis indicated Log relative risk of respiratory hospitalization. The blue lines were the 95% CI. All models were adjusted for time, temperature, relative humidity, weekend and public holiday.)

Table S1. The percent change and 95% CI for hospitalization due to respiratory disease associated with a 10-ug/m3 increases in air pollutants concentrations by gender and age in single-pollutant models in Taiyuan, China.

Pollutants	Male	Female	Age < 65 years	Age ≥ 65 years
$PM_{2.5}$				
lag0	0.364(−0.012,0.741)	0.446(0.063,0.830) *	0.403(0.046,0.761) *	0.370(−0.140,0.882)
lag1	0.350(0.004,0.697) *	0.331(−0.022,0.686)	0.411(0.081,0.741) *	0.137(−0.329,0.605)
lag2	0.116(−0.220,0.453)	0.271(−0.071,0.613)	0.280(−0.039,0.600)	−0.104(−0.555,0.348)
lag3	−0.014(−0.349,0.323)	0.047(−0.295,0.391)	0.153(−0.167,0.473)	−0.379(−0.830,0.074)
lag4	−0.043(−0.380,0.294)	0.046(−0.297,0.391)	0.145(−0.175,0.467)	−0.439(−0.891,0.016)
lag5	−0.035(−0.374,0.305)	0.132(−0.213,0.478)	0.116(−0.207,0.439)	−0.198(−0.655,0.260)
lag01	0.496(0.071,0.922) *	0.535(0.101,0.971) *	0.567(0.163,0.972) **	0.338(−0.237,0.915)
lag02	0.482(0.011,0.954) *	0.615(0.135,1.098) *	0.648(0.201,1.097) **	0.205(−0.430,0.843)
lag03	0.429(−0.088,0.948)	0.587(0.059,1.117) *	0.679(0.188,1.173) **	−0.043(−0.739,0.659)
lag04	0.383(−0.178,0.949)	0.584(0.009,1.162) *	0.728(0.194,1.265) **	−0.290(−1.047,0.474)
lag05	0.356(−0.251,0.967)	0.643(0.021,1.269) *	0.775(0.199,1.355) **	−0.389(−1.207,0.436)
PM_{10}				
lag0	0.245(0.004,0.487) *	0.273(−0.261,0.810) *	0.252(0.022,0.482) *	0.270(−0.055,0.595)
lag1	0.251(0.018,0.485) *	0.202(−0.038,0.442)	0.292(0.068,0.515) *	0.056(−0.258,0.371)
lag2	0.129(−0.105,0.364)	0.209(−0.030,0.449)	0.238(0.015,0.462) *	−0.053(−0.369,0.264)
lag3	0.042(−0.193,0.276)	0.093(−0.147,0.333)	0.158(−0.066,0.381)	−0.200(−0.516,0.116)
lag4	0.063(−0.169,0.295)	0.094(−0.144,0.332)	0.174(−0.047,0.396)	−0.203(−0.514,0.109)
lag5	0.029(−0.204,0.263)	0.122(−0.116,0.361)	0.123(−0.099,0.346)	−0.09(−0.403,0.223)
lag01	0.331(0.057,0.606) *	0.315(0.034,0.597) *	0.363(0.101,0.625) **	0.210(−0.158,0.580)
lag02	0.349(0.043,0.657) *	0.382(0.068,0.698) *	0.438(0.146,0.731) **	0.145(−0.268,0.560)
lag03	0.338(0.001,0.676) *	0.394(0.049,0.741) *	0.478(0.157,0.800) **	0.026(−0.428,0.482)
lag04	0.347(−0.017,0.712)	0.415(0.042,0.790) *	0.534(0.187,0.882) **	−0.078(−0.567,0.414)
lag05	0.347(−0.043,0.738)	0.457(0.056,0.859) *	0.571(0.199,0.944) **	−0.117(−0.641,0.410)
SO_2				
lag0	0.362(0.042,0.684) *	0.486(0.158,0.814) **	0.399(0.095,0.705) *	0.460(0.023,0.899) *
lag1	0.460(0.149,0.772) **	0.344(0.026,0.664) *	0.407(0.110,0.704) **	0.442(0.021,0.865) *
lag2	0.255(−0.047,0.557)	0.400(0.093,0.709) *	0.338(0.050,0.627) *	0.247(−0.157,0.652)
lag3	0.058(−0.242,0.358)	0.190(−0.115,0.497)	0.175(−0.111,0.462)	−0.048(−0.448,0.353)
lag4	−0.033(−0.333,0.268)	0.019(−0.287,0.327)	0.128(−0.159,0.415)	−0.391(−0.791,0.011)
lag5	−0.063(−0.364,0.238)	−0.07(−0.377,0.238)	0.000(−0.287,0.288)	−0.240(−0.640,0.162)
lag01	0.621(0.234,1.009) **	0.627(0.231,1.025) **	0.605(0.238,0.973) **	0.692(0.162,1.224) *
lag02	0.735(0.293,1.180) **	0.845(0.391,1.301) ***	0.783(0.363,1.204) ***	0.781(0.176,1.390) *
lag03	0.715(0.229,1.203) **	0.900(0.403,1.400) ***	0.843(0.383,1.305) ***	0.674(0.014,1.338) *
lag04	0.649(0.128,1.172) *	0.848(0.314,1.385) **	0.875(0.383,1.370) ***	0.370(−0.337,1.082)
lag05	0.587(0.031,1.145) *	0.772(0.201,1.347) **	0.845(0.321,1.373) **	0.206(−0.548,0.966)
NO_2				
lag0	1.526(0.442,2.620) **	2.029(0.900,3.170) ***	1.408(0.373,2.454) **	2.662(1.172,4.173) ***
lag1	1.049(−0.013,2.122)	0.968(−0.119,2.068)	0.883(−0.133,1.909)	1.431(0.006,2.877) *
lag2	1.120(0.079,2.171) *	1.406(0.337,2.485) **	1.299(0.300,2.307) *	1.062(−0.320,2.464)
lag3	0.737(−0.303,1.788)	1.407(0.336,2.488) *	1.134(0.134,2.144) *	0.654(−0.727,2.054)
lag4	0.234(−0.804,1.282)	0.530(−0.536,1.608)	0.724(−0.274,1.733)	−0.668(−2.032,0.715)
lag5	0.187(−0.849,1.234)	0.281(−0.783,1.356)	0.379(−0.617,1.385)	−0.157(−1.528,1.233)
lag01	1.741(0.490,3.008) **	2.022(0.727,3.334) **	1.548(0.352,2.757) *	2.783(1.071,4.524) **
lag02	2.119(0.720,3.539) **	2.522(1.077,3.987) ***	2.061(0.723,3.418) **	2.936(1.037,4.871) **
lag03	2.311(0.775,3.871) **	3.045(1.458,4.658) ***	2.476(1.004,3.970) ***	2.988(0.916,5.102) **
lag04	2.304(0.636,4.001) **	3.144(1.419,4.898) ***	2.719(1.117,4.347) ***	2.437(0.201,4.723) *
lag05	2.344(0.538,4.182) *	3.216(1.347,5.119) ***	2.854(1.117,4.620) **	2.293(−0.120,4.764)
O_3				
lag0	0.099(−0.456,0.658)	0.059(−0.521,0.642)	0.05(−0.488,0.591)	0.273(−0.484,1.036)
lag1	0.312(−0.236,0.862)	0.428(−0.143,1.002)	0.390(−0.141,0.923)	0.313(−0.417,1.048)
lag2	0.023(−0.515,0.565)	−0.115(−0.676,0.449)	−0.004(−0.526,0.521)	−0.083(−0.794,0.633)
lag3	−0.232(−0.770,0.309)	−0.161(−0.722,0.404)	−0.270(−0.791,0.255)	−0.017(−0.730,0.702)
lag4	−0.120(−0.657,0.420)	0.003(−0.557,0.566)	−0.169(−0.689,0.354)	0.207(−0.505,0.924)
lag5	0.418(−0.122,0.960)	0.407(−0.155,0.972)	0.391(−0.131,0.916)	0.495(−0.220,1.216)
lag01	0.289(−0.360,0.943)	0.344(−0.333,1.027)	0.311(−0.318,0.944)	0.421(−0.458,1.309)
lag02	0.250(−0.468,0.972)	0.211(−0.538,0.965)	0.251(−0.444,0.950)	0.279(−0.683,1.251)
lag03	0.098(−0.676,0.879)	0.102(−0.707,0.918)	0.080(−0.671,0.836)	0.229(−0.805,1.274)
lag04	0.031(−0.796,0.865)	0.095(−0.771,0.968)	−0.009(−0.811,0.799)	0.313(−0.791,1.429)
lag05	0.220(−0.661,1.109)	0.277(−0.645,1.207)	0.170(−0.685,1.032)	0.530(−0.645,1.719)

Table S2. Sensitivity analysis for health effects of ambient air pollution on respiratory hospitalization by changing df=2-5 per year of time, temperature and relative humidity in Taiyuan, China (health effects were showed as percent changes and 95% CI with per 10-ug/m3 increment in air pollutants).

Variables	$PM_{2.5}$	PM_{10}	SO_2	NO_2
Time				
df=2/year	0.407(0.066,0.750) *	0.242(0.020,0.464) *	0.422(0.134,0.710) **	1.225(0.250,2.208) **
df=3/year	0.424(0.080,0.770) *	0.278(0.055,0.501) *	0.485(0.195,0.776) **	1.755(0.745,2.776) **
df=4/year [a]	0.397(0.045,0.751) *	0.257(0.031,0.484) *	0.407(0.106,0.708) **	1.682(0.664,2.711) **
df=5/year	0.296(0.093,0.744) *	0.216(0.024,0.430) *	0.360(0.103,0.719) **	1.350(0.346,2.364) **
Average temperature				
df=2	0.397(0.045,0.751) *	0.257(0.031,0.484) *	0.407(0.106,0.708) **	1.682(0.664,2.711) **
df=3 [a]	0.397(0.045,0.751) *	0.257(0.031,0.484) *	0.407(0.106,0.708) **	1.682(0.664,2.711) **
df=4	0.369(0.019,0.721) *	0.232(0.007,0.458) *	0.416(0.118,0.715) **	1.504(0.486,2.531) **
df=5	0.372(0.022,0.723) *	0.232(0.007,0.458) *	0.413(0.115,0.713) **	1.493(0.477,2.519) **
Relative humidity				
df=2	0.397(0.045,0.751) *	0.257(0.031,0.484) *	0.407(0.106,0.708) **	1.682(0.664,2.711) **
df=3 [a]	0.397(0.045,0.751) *	0.257(0.031,0.484) *	0.407(0.106,0.708) **	1.682(0.664,2.711) **
df=4	0.397(0.045,0.751) *	0.257(0.031,0.484) *	0.407(0.106,0.708) **	1.684(0.665,2.714) **
df=5	0.397(0.045,0.751) *	0.257(0.031,0.484) *	0.407(0.106,0.708) **	1.683(0.665,2.712) **

a represented the df used in the present study. * $p < 0.05$, ** $p < 0.01$, *** $p < 0.001$, O_3 wasn't included in the sensitivity analysis because the effects on respiratory hospitalization had no statistical significance.
